# B-HIT - A Tool for Harvesting and Indexing Biodiversity Data

**DOI:** 10.1371/journal.pone.0142240

**Published:** 2015-11-06

**Authors:** Patricia Kelbert, Gabriele Droege, Katharine Barker, Kyle Braak, E. Margaret Cawsey, Jonathan Coddington, Tim Robertson, Jamie Whitacre, Anton Güntsch

**Affiliations:** 1 Botanic Garden and Botanical Museum Berlin-Dahlem, Freie Universität Berlin, Berlin, Germany; 2 National Museum of Natural History, Smithsonian Institution, Washington DC, United States of America; 3 Global Biodiversity Information Facility, Copenhagen, Denmark; 4 Australian National Wildlife Collection, CSIRO National Research Collections Australia, Canberra, Australia; Università di Genova, ITALY

## Abstract

With the rapidly growing number of data publishers, the process of harvesting and indexing information to offer advanced search and discovery becomes a critical bottleneck in globally distributed primary biodiversity data infrastructures. The Global Biodiversity Information Facility (GBIF) implemented a Harvesting and Indexing Toolkit (HIT), which largely automates data harvesting activities for hundreds of collection and observational data providers. The team of the Botanic Garden and Botanical Museum Berlin-Dahlem has extended this well-established system with a range of additional functions, including improved processing of multiple taxon identifications, the ability to represent associations between specimen and observation units, new data quality control and new reporting capabilities. The open source software B-HIT can be freely installed and used for setting up thematic networks serving the demands of particular user groups.

## Introduction

Over the last 15 years, The Global Biodiversity Information Facility (GBIF [1]) and related initiatives such as BioCASe [2] and SpeciesLink [3] have built a capable global information infrastructure providing instant and unified access to a large number of biological collection and observational data [4]. By October 2015, more than 577 million primary information records, each documenting a particular collection or observational event, are accessible through a range of web portals and service APIs, providing an indispensable basis for biodiversity related data-driven research. In their initial development phase, biodiversity data networks such as ENHSIN [5], SpeciesAnalyst [6] and GeoCASE [7] were conceived as entirely distributed systems with user queries and responses simultaneously propagated through the entire network each time they were requested. This approach quickly proved inadequate for larger provider numbers and user requests. As a consequence, biodiversity networks started to develop central indexing databases. These databases aimed to provide complete inventories of data provider nodes, and cached a limited set of data elements considered sufficient to provide responses for the majority of user requests without having to forward them to the data providers. The availability of index databases dramatically improved both the stability and responsiveness of biodiversity data services and portals. It additionally removed the need for a user to understand the various protocols necessary to communicate with data holding institutions. However, keeping the indexes up-to-date and consistent requires additional software components that must provide mechanisms for effective harvesting, indexing, data storage and data quality control. The GBIF Harvesting and Indexing Toolkit (GBIF-HIT [8]) provides an integrated web-based harvesting platform for collection and observation data. The GBIF-HIT is capable of processing the standard protocols for biodiversity data retrieval such as DiGiR (Distributed Generic Information Retrieval [9]), BioCASe-Protocol [10] and TAPIR (TDWG Access Protocol for Information Retrieval [11]), as well as the relevant content standards including Darwin Core [12] and ABCD (Access to Biological Collection Data) [13].

Initially developed for building and maintaining the GBIF index database, the GBIF-HIT is also used in a range of international biodiversity networks such as GBIF country nodes and OpenUp! [14]. In the context of the German Biodiversity Network of the Humboldt-Ring (BiNHum, http://wiki.binhum.net/web/) and the Global Genome Biodiversity Network (GGBN, http://www.ggbn.org, [15]), we have added a set of functionalities making this toolkit even more flexible and generically usable within biodiversity special interest networks. The new functions include the capability of processing multiple identifications (identification histories) without record duplication, as well as specific multimedia information, associated collection and observational units (e.g. pathogenic fungi on plants, DNA-tissue-specimen relations), molecular data (GGBN Data Standard, http://terms.tdwg.org/wiki/GGBN_Data_Standard), and environmental data. Furthermore, a set of new data quality tests and data quality reports has been added. The new HIT, now called B-HIT, has been successfully deployed as a part of the central network components of BiNHum and GGBN. The software source code including documentation and installation guidelines is freely and openly available under Apache license Version 2.0 from http://wiki.bgbm.org/bhit and http://ww2.biocase.org/svn/.

## Materials and Methods

### The original HIT

Harvesting and aggregating data from a large number of providers is a complex process, in particular when several access protocols, versions, data standards and their variations must be supported. In addition, the harvester must deal with variable availability of provider services, ranging from highly reliable installations to unstable providers with frequent or long downtimes.

The GBIF-HIT was designed by GBIF with the aim to streamline the harvesting process in the rapidly growing international biodiversity data network. This was achieved by combining a registry of endpoints to harvest and a scheduler to automate indexing, and by integrating application runtime logs to assist a data manager with diagnosing issues. GBIF-HIT is an open source Java web-application, with graphical user-interface (GUI) that facilitates management and monitoring of the data harvesting process and the construction of specific index databases, providing a unified data representation for all primary records.

#### Supported protocols and schemas

In its original version, the GBIF-HIT supported the two most commonly used biodiversity standards: Darwin Core and ABCD, and their corresponding exchange protocols and middleware BioCASe, DiGIR and TAPIR. Additional support has also been added for the Darwin Core Archive (DwC-A), making data available for upload through a single dump by using a separate tool (e.g. the Integrated Publishing Toolkit (IPT), http://www.gbif.org/ipt [16]). These middleware components are installed and configured on each server that provides data to biodiversity networks by wrapping communications between the host databases and the data aggregators.

#### Harvesting process

**Fig 1:**
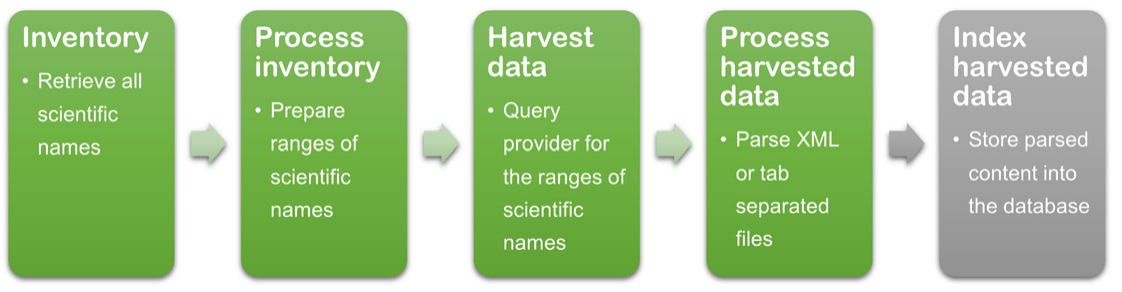
GBIF-HIT Harvesting process. It consists of 4 major steps that have to be executed after each update of a datasource. The harvested data is eventually parsed and stored into the database.

The harvesting of the GBIF-HIT was broken down into four major operations: 1) inventorying, 2) processing inventory and creating name ranges, 3) harvesting, and 4) processing and indexing harvested data. These operations were followed by indexing the harvested data (Fig 1). The inventory operation consisted of requesting a list of all scientific names that are contained within a specific dataset. Name ranges composed of lower and upper scientific names were generated. The harvesting operation consisted of processing these name ranges and dispatching a search request for all records greater than or equal to the lower name range, and less than or equal to the upper name range. The harvested records were then processed as follows: the search responses are collected, parsed (using editable mapping files to identify which elements are to be parsed), and the parsed values are eventually written to file(s). The final operation, indexing, synchronised the processed harvested records with a data store such as a MySQL database.

### Requirements

The original GBIF-HIT did not address some valuable information. One example is the relationships between specimens, such as a host-parasite or a tissue-specimen association, which can link to one or more supplemental levels, as several DNA samples or sequences can be extracted from a single tissue and underlying voucher specimens can be deposited in different institutions. These complexities imply that relations between all associated records for a certain dataset must be stored. Another example is the need to harvest permit and loan details, as a result of the Nagoya Protocol [17], which entered into force on 12^th^ October 2014. Finally, users require the ability to search for multiple identifications (historical changes or corrections) for a single record; the current GBIF-HIT creates distinct records (occurrences) for every identification. Users might also require the ability to harvest only a subset of a dataset, based on a list of record IDs.

Datasets rich in multimedia data and metadata, e.g. sound files or images, are increasingly common and important (the 2 million records from the BiNHum partners contain more than 330.000 distinct multimedia files, and new objects get digitised every day). Therefore the team of the Botanic Garden and Botanical Museum (BGBM) extended the existing GBIF-HIT multimedia functionality with additional parameters, such as multimedia object size, copyright or licenses. The new HIT version also needed to support the recently developed new output format for ABCD records (ABCD archive, available since BioCASe Provider Software v3.4, http://wiki.bgbm.org/bps/index.php/VersionHistory), which accommodates large datasets. Lastly, based on the data provided by the project partners, specific quality controls had to be performed.

The architecture and concept of the original GBIF-HIT was ideal for harvesting small and medium scale data. To implement the new functionalities listed above, we decided to develop a new version of the Harvesting and Indexing Toolkit (B-HIT), while preserving as much of the original architecture as possible. However, the new requirements did necessitate definition of a new underlying MySQL database schema.

## Results

### Harvesting

The inventory method of the newly developed B-HIT has changed. In order to avoid missing records and prevent encoding problems it now looks for the mandatory UnitID (record identifier of ABCD, also known as CatalogNumber in Darwin Core) rather than the non-mandatory scientific name. B-HIT can process records delivered in ABCD 2.06, ABCD 2.1, ABCDEFG (Access to Biological Collection Data—Extended for Geosciences [18]), ABCDGGBN (DNA extension), ABCD-Archives, Darwin Core, and Darwin Core Archive [19], (Tables 1 and 2). ABCD 1.2 is now deprecated, as it is no longer used by BioCASe providers.

**Table 1:** ABCD versions supported by the GBIF-HIT and B-HIT.

	ABCD version						
	1.2	2.06	2.1	EFG	GGBN	GGBN Enviro	ABCD—Archive
**GBIF-HIT**	X	X					
**B-HIT**		X	X	X	X	X	X

Supported ABCD versions are marked with an X.

**Table 2:** Darwin Core versions supported by the GBIF-HIT and B-HIT.

	Darwin Core (DwC) version		
	DwC 1.0, 1.4, 1.4-Geospatial, 1.4-Curatorial, MaNIS 1.0, MaNIS 1.21	DwC Archive	DwC GGBN
**GBIF-HIT**	X	X	
**B-HIT**	X	X	X

Supported DwC versions are marked with an X.

### Index Database

**Fig 2:**
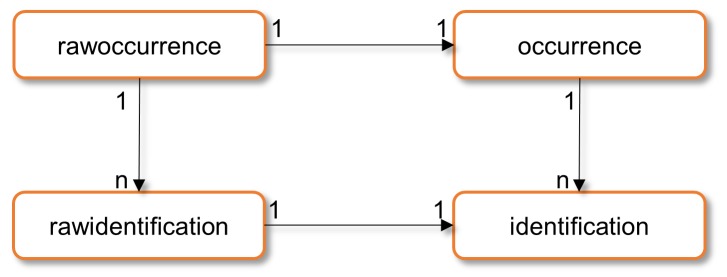
Principal model of raw and improved data in the B-HIT database. Corresponding raw and improved table (i.e. identification from the provider and improved identification) are linked through a 1–1 relation. Multiple identifications can be associated to a single record and are therefore linked through a 1-n relation with the (raw) occurrence table.

The database schema underwent significant extension and adaptation to support several 1-to-many relationships within a single record, such as multiple identifications, multimedia urls and associations. In its current version (October 2015), the database consists of 37 tables, divided into a raw data block and an improved data block (Fig 2). All original values harvested from providers are kept and stored. In addition, the improved data block comprises tables with similar structure, but holding cleaned and improved data after quality control. This principal architecture of B-HIT also allows data portal developers to choose between raw and cleaned data for search and display issues.

### New Features

**Fig 3:**
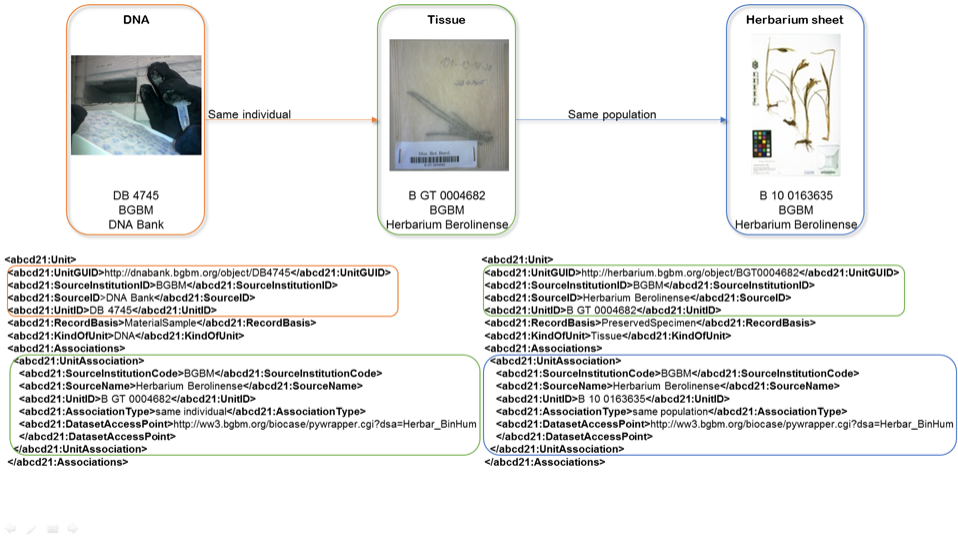
Representation of associated units in ABCD 2.1. A DNA Sample with the triple ID “DB 4745—BGBM—DNA Bank” is associated to a tissue (triple ID “B GT 0004682—BGBM—Herbarium Berolinense”). This tissue is associated to a herbarium sheet (triple ID “B 10 0163635—BGBM—Herbarium Berolinense”). The associated dataset access point and triple ID make it possible to retrieve each record.

Supporting and enabling indexing of associations between single records is one of the key new features of B-HIT. Both ABCD and Darwin Core include terms to define and describe a relation between two records (Fig 3). Because globally unique identifiers are not yet in place at each provider, B-HIT uses the established GBIF triple ID (UnitID—SourceID—SourceName for ABCD and CatalogNumber—InstitutionCode—CollectionCode for Darwin Core) and, if available, a GUID (Globally Unique Identifier). Second, the terms describing a relation are free text in both standards. Therefore, the community using these terms must agree on a convention for using them, which both BiNHum and GGBN have done by choosing a set of values to be used as recommended vocabulary.

B-HIT can harvest and index records having not only botanical, zoological and bacterial names, but also scientific names of minerals. These are then tagged specifically in the underlying database, so that the corresponding records can be handled separately for quality controls or for a future output in a data portal. These non-biological names are provided by ABCD, not by Darwin Core. Stratigraphic terms are indexed for both ABCD and Darwin Core records and can be matched for cleaning against any standardised stratigraphy list.

**Fig 4:**
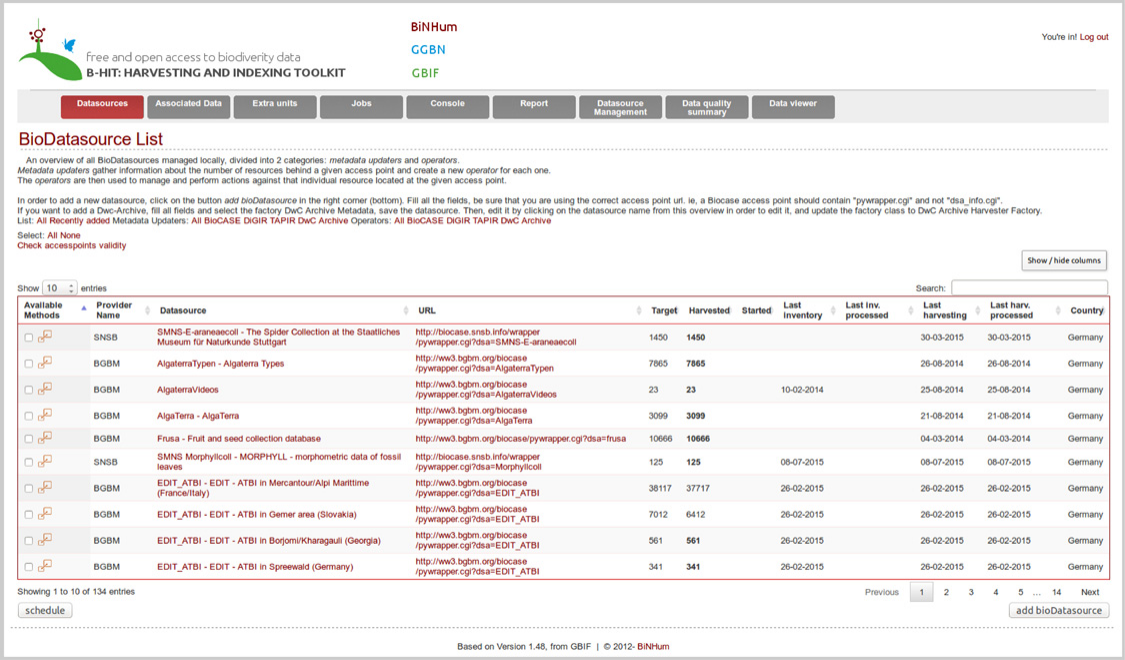
Web interface of B-HIT. This extended user-interface makes it possible to gain access to the new functionalities (i.e. Associated Datasource Harvesting, Data quality, Datasource Management) through a series of tabs.

Every triple ID occurring in a record (both main triple ID and associated triple ID), as well as their relations, are stored. This enables portal developers to get information on all parent and grandparent entries for a single record. B-HIT checks every associated triple ID for its existence and availability at a respective provider. B-HIT is capable of preparing the data source metadata—such as name, access point, collection code, institution code—based on the relationship information stored by the main dataset. A new tab has been added into the GUI, dedicated to a user-friendly handling of this special category of datasets (Fig 4). Missing associations are checked: if some records should be linked to external or internal datasets, B-HIT will automatically look for the presence of these associated datasets and the corresponding records in the database. Specific functions are set for the associated data sources, such as harvesting the list of missing units only and processing these units; harvesting their sibling units and processing them. If associated datasets or units are still missing after these operations are run, the main data source will be associated with a special mark on the overview.

Another feature has been added for DwC-A providers to fully support associations between records. The archive is downloaded in whole and the required siblings and parent units are extracted. As the archives can be very large (several Gigabytes), a supplemental step was implemented: the B-HIT now reduces the large file to a smaller archive, by only copying the records required for the associations. The size of the new archive is dramatically shrunk to a few Kilobytes, and its reuse for e.g. portal display is facilitated.

Extended quality checks and data enrichments have been implemented into B-HIT and its user front-end to allow provision of feedback on data quality to data providers. A quality management tab has been added to launch the tests and view the outcomes of these tests. These algorithms check e.g. geographic values (country names vs. ISO-codes, coordinates), date formats and scientific names validity. Furthermore, a separate tool, compatible with B-HIT, has been developed to search for names against individual datasets within the GBIF Checklist Bank (https://github.com/gbif/checklistbank), in order to get both accepted names for synonyms and higher taxa. Every quality test generates logs (with test name, original value, suggested correction, explanation and record IDs (i.e. UnitIDs)), that can be exported through the user-interface as text files with tab-separated values, creating one file per dataset and per test (Table 3). Quality tests will generate n x m files (n = number of datasources, m = number of tests run) in the worst case scenario. The system will only extract problematic rows, i.e. when a test failed or generated a warning. These reports can be sent to dataset curators. In the case of the BiNHum project, these files were sent to providers who then used them to improve the quality of their data.

**Table 3:** Export subset of the quality logs.

Test	Original value (countryname—ISOcode)	New value (countryname—ISOcode)	Suggestion or log	UnitIDs
**country**	none-CH	Switzerland-CH	extracted country from gathering area	Bridel-1-512 Bridel-1-576 [..]
**country**	none-none	United States-US	extracted country from locality	Bridel-1-525 Bridel-1-898 [..]
**country**	none-MG	Madagascar-MG	extracted country from locality	Bridel-1-206 Bridel-1-359
**country**	Slovak Republic-none	Slovakia-SK	countryname replaced Slovak Republic by Slovakia	M-0136500-550428-132827 [..]
**country**	Bayern-none	Germany-DE	countryname replaced Bayern by Germany	ZSM-A-20032864 / 604358 / 487654

The dedicated tables, for each kind of test, store the test name, the value from the provider, the improved value, and a brief explanation. The list of concerned records is also saved in the quality tables for helping the provider to find and correct its data.

## Discussion

B-HIT, an evolution of the GBIF Harvesting and Indexing Toolkit, provides a range of new functionalities, including the ability to represent and process associations between specimen and observational data, as well as new data quality tests and reporting capabilities, helping primary information providers to improve their data. The system deals with well-established biodiversity informatics access protocols and data exchange standards (ABCD and Darwin Core) and can be extended to support upcoming versions or other standards. B-HIT is ideally suited to set up special interest networks with a specific thematic scope in a very effective way. The MySQL database of B-HIT can be used as a main index database, but can also be combined with a search platform (e.g. a SOLR instance [20])—as being done for both BiNHum and GGBN to speed up queries. The database structure, storing both raw and improved data, enables a high flexibility for web-portal developments.

The original codebase of the GBIF Harvesting and Indexing Toolkit is no longer actively worked on by GBIF. As the GBIF network grew, GBIF switched to a new indexing solution built around data streaming and Hadoop technologies for large scale indexing. However, the B-HIT technology still plays an important role for effectively setting up smaller “special interest” networks with a particular geographic, taxonomic, or thematic focus.

In order for the GBIF registry to initiate data crawls, a messaging system was put into place in the IPT. A similar messaging and updating system with project specific registries is also planned for BioCASe and the B-HIT.

B-HIT is already successfully used as the central data harvester for both the German Biodiversity Network of the Humboldt Ring (BiNHum) and the Global Genome Biodiversity Network (GGBN). Additional deployments of the software are planned for several BioCASe-based special interest networks, as well as for smaller integrated portals for institutions providing access across different collection types (e.g. preserved, living, DNA, seed bank, multimedia). A portal using B-HIT for aggregating specimen data from distributed herbaria contributing to the World Flora Online [21] is under construction.

The software can also be used to share non-biodiversity data. A portal with geological data can easily be supported, as B-HIT can handle scientific names for e.g. minerals and allows indexing and searching for geological eras. Specific quality controls for these domains could easily be added to the existing tests (e.g. translation or control vocabulary for stratigraphic terms).

Another attractive perspective for future B-HIT extensions is its integration into research workflows as a mechanism for compiling data sets required for occurrence-data based "in-silico" experiments [22]. Today's workflow environments usually rely on occurrence data being highly organised through services of the global biodiversity data infrastructures. Integration of additional not yet fully integrated resources represents a big hurdle for scientists. B-HIT could be extended towards a self-contained package for harvesting and integrating data from existing aggregators, individual biodiversity data services, and local data packages that have not yet been published, thus providing a powerful and easy-to-use solution for integrating data across different publication levels.

Long term support and codebase maintenance is provided by BGBM. B-HIT is included in the BGBM codebase repository (http://ww2.biocase.org/svn/). We appreciate any feedback to improve the system.
